# Women’s Empowerment as a Mitigating Factor for Improved Antenatal Care Quality despite Impact of 2014 Ebola Outbreak in Guinea

**DOI:** 10.3390/ijerph17218172

**Published:** 2020-11-05

**Authors:** Laura K. Merrell, Sarah R. Blackstone

**Affiliations:** Department of Health Sciences, James Madison University, Harrisonburg, VA 22807, USA; blackssr@jmu.edu

**Keywords:** women’s empowerment, antenatal care, Guinea, demographic health survey

## Abstract

Improving maternal outcomes and reducing pregnancy morbidity and mortality are critical public health goals. The provision of quality antenatal care (ANC) is one method of doing so. Increasing women’s empowerment is associated with positive women’s health outcomes, including the adequate timing and amount of ANC use. However, little is known about the relationship between women’s empowerment and quality ANC care. Despite a history of political instability, low women’s equality and poor maternal health, the Republic of Guinea has committed to improving the status of women and access to health. However, the 2014 Ebola outbreak may have had a negative impact on achieving these goals. This study sought to examine factors in the relationship between women’s empowerment and the receipt of quality ANC (indicated by the number of health components) within the context of the Ebola outbreak. This study conducted multiple logistic regressions examining associations between covariates and the number of ANC components received using data from the 2012 and 2018 Guinea Demographic Health Surveys. Several aspects of women’s empowerment (healthcare decision-making, literacy/access to magazines, monogamous relationship status, contraceptive use, socio-economic status/employment) were significantly linked with the receipt of a greater number of ANC components, highlighting the importance of women’s empowerment in accessing quality maternity care.

## 1. Introduction

Empowerment has many definitions within the literature, but almost all recognize empowerment as a process through which an individual possesses the agency and resources to expand their ability to make and act upon strategic life choices, in a context wherein this ability was previously denied to them [1,2]. Increasing women’s empowerment has been an important multidimensional global developmental goal for several decades. Increased empowerment is associated with many improved women’s health outcomes, which are most often researched in sexual and reproductive health contexts [3,4,5]. However, relatively little attention has been focused on the relationship between empowerment and pregnancy outcomes.

Complications during pregnancy, childbirth, and the post-natal period are leading causes of death among women, particularly in middle- and low-income countries [6]. Global estimates indicate there were approximately 300,000 maternal deaths in 2017, 35% lower than in 2000 [7]. However, these improvements have not equally benefitted all women. Whereas the global maternal mortality rate (MMR) rate is 211 per 100,000 live births, Sub-Saharan Africa was estimated to have 542 maternal deaths per 100,000 live births in 2017 (66% of all maternal deaths) [7].

Quality antenatal care (ANC) is one method of preventing maternal morbidity and mortality. ANC is defined as the care provided by skilled healthcare professionals to pregnant women and girls to ensure the best health conditions before, during, and after childbirth, and includes risk identification, the prevention and management of pregnancy-related or concurrent diseases, and health education and promotion [8]. ANC reduces maternal and perinatal morbidity and mortality directly through the detection and treatment of pregnancy-related complications, and indirectly through the identification of at-risk populations, ensuring referral to other means of appropriate care and the prevention and management of concurrent diseases [9,10]. 

ANC quality has been measured by three indicators: (1) timing of entrance into ANC; (2) number of ANC visits; (3) the inclusion of all recommended components of care [11]. Historically, the literature looking at the quality of ANC care has focused on the number and timing of ANC visits. Whereas prior to 2016, the World Health Organization recommended a minimum of four ANC visits during pregnancy, newer guidelines recommend a minimum of eight ANC contacts [8]. Globally, approximately 86% of pregnant women access ANC with skilled health personnel at least once, and only 65% receive at least four ANC visits [8]. While ANC contact has increased in Sub-Saharan Africa over the past fifteen years, only 52% of pregnant women receive four ANC visits [12]. 

The early initiation of ANC, preferably in the first trimester, is also important to quality care. Receiving care early in the pregnancy allows for the prompt identification of potential problems [13] and encourages women to adhere to pregnancy recommendations earlier [14,15,16]. While these factors are important components of care, many studies in low- to middle-income countries have found a positive yet weak association between ANC contact and timing and maternal outcomes, including maternal complications [17], mortality [18], stillbirths [19] and low birth weight [20]. 

Therefore, recent research has looked at the provision of the recommended components of care as further evidence of the quality of ANC [21]. The recommended components of ANC include provision or iron supplementation, blood and urine samples, blood pressure measurements, at least two tetanus toxoid injections, intestinal parasite drugs, and health education [22]. All components play a role in the provision of quality care, promoting health during and after a woman’s pregnancy [23,24,25]. Further declines have been seen in maternal and infant mortality when women receive care from a skilled birth attendant (SBA), such as a nurse, doctor, or physician’s assistant [24,25]. Numerous studies have found that both structural (material, human, and financial resources) and process (what is actually done/covered in the provision of care) attributes impact the quality of ANC [18,22,26]. Further, the new 2016 WHO ANC guidelines aim to not only reduce maternal morbidity and mortality as an outcome of quality ANC, but also improve women’s experiences of care. Achieving positive motherhood includes improving maternal self-esteem and autonomy [27]. 

### 1.1. ANC and Women’s Empowerment

The barriers to accessing and utilizing ANC services are well-documented and include cost, distance, transportation and lack of knowledge [26,28,29]. However, women’s lack of empowerment may also negatively affect the utilization of ANC services and the quality of care [22,30,31]. Women often lack their own financial resources or the decision-making power to allocate them to healthcare [32,33]. Decisions about whether, and for what purpose, women utilize healthcare services may be made exclusively by the men in their lives (husband/father/son) [34,35]. Even if a woman does access ANC, her limited personal financial resources and decision-making autonomy may impact if she is able to implement recommended practices at home [36]. 

Alternately, studies have found empowerment to be positively associated with increased pregnancy healthcare seeking [37,38], skilled delivery attendance [37,39] and the use of modern contraceptive methods [5,40], resulting in lower infant mortality [41]. While there are many ways to conceptualize women’s empowerment, previous studies have conceptualized empowerment as it relates to pregnancy outcomes, such as via health decision-making [42], attitudes toward intimate partner violence [13], education [13,43], employment [44,45] wealth [22], access to media [22] and contraceptive use [31].

The importance of women’s empowerment has been well documented for many health outcomes, however its relationship with the quality of ANC, specifically the number of components of care received, remains understudied despite better pregnancy outcomes being linked with the receipt of an appropriate number of ANC health components [30]. Given that there have been improvements in ANC contact and the timing for ANC initiation, yet there are still large gaps in maternal mortality and pregnancy outcomes, there is compelling reason to explore factors influencing the quality of the care pregnant women are receiving. 

### 1.2. Republic of Guinea

The Republic of Guinea, a West-African country and former French colony, has experienced significant social and political turmoil since its independence. Previously considered a ‘high alert’ state according to the Fragile States Index, it has shown consistent improvement over the last decade [46]. Women in Guinea face numerous threats to health, including HIV/AIDS, food insecurity, and high infant mortality. As of 2017, Guinea is estimated to have ‘Very High MMR’, with 576 maternal deaths per 100,000 live births (14th highest) [7]. However, at the same time, this represents a 44% reduction in maternal mortality in the country since 2000 [7]. Further, women in Guinea experience significant gender inequity. It is the fourth most gender-unequal country in Africa [47]. Only 66% of women (compared to 78% of men) participate in the paid labor market. Life expectancy, expected years of schooling, and per capita GNI are all lower compared to men [48]. According to the Human Development Report, Guinea scored in the bottom third for several indicators of women’s empowerment. 

However, the country has committed to addressing women’s empowerment, and taken several positive steps. In 2015, the government acknowledged that education and employment opportunities are male-dominated and committed to creating better education and work opportunities for women [49]. In 2016, the World Bank and the Sexual Violence Research Initiative donated USD 3.5 million to be spent in different African countries to address violence against women, resulting in several campaigns in Guinea to address gender inequality [50]. In May 2020, The World Bank earmarked an additional USD 60 million for efforts to build human capital among women in Guinea, including investment in activities that target adolescent girls to improve sexual and reproductive health knowledge and remain in secondary education, create an enabling environment for economic opportunity, and improve legal frameworks that improve women’s rights to health and education [51]. 

During the 2014 Ebola outbreak, Guinea was one of the countries most heavily affected. Guinea is estimated to have had over 3800 suspected cases, which resulted in 2544 confirmed deaths [52]. Numerous studies have demonstrated the negative impact the outbreak had on access to maternal and child healthcare and MCH outcomes [53,54,55]. During the 2014 outbreak, there was a decrease in hospital deliveries and deliveries by skilled birth attendants [53,54]. Further, the proportion of women receiving ANC post-outbreak decreased from estimates pre-outbreak, as did the uptake of child vaccinations [55]. 

Given the work toward progressing women’s empowerment in Guinea and the high maternal mortality ratio, the purpose of this study is to examine factors associated with quality of ANC received (as indicated by number of health components received) in Guinea during two time periods. This study fills a gap in the literature by exploring the relationship between different aspects of women’s empowerment and ANC. Specifically, this study seeks to describe whether women’s empowerment may be associated with improvements in access to quality ANC (measured as components rather than timing and number of ANC visits). Given the work that Guinea has done on women’s empowerment, we expect that more women will receive the appropriate number of ANC components in 2018. We also predict that a variety of women’s empowerment attributes, including health decision-making, attitudes toward intimate partner violence (IPV), contraceptive use, media exposure, employment and education, will be associated with the receipt of better-quality care. 

While the aim of this study does not specifically look at the impact of the 2014 Ebola outbreak on ANC receipt, we include a discussion of it as a contextualizing factor. The impact of the outbreak is likely to have had long-term impacts on the healthcare system, including access to maternal care such as ANC. Given that the participants included in this analysis were pregnant only two years after the outbreak ended, it is not clear if the consequences of the outbreak will be seen in reductions in quality ANC over the two time periods.

## 2. Materials and Methods 

### 2.1. Data Sources

This study used data from the 2012 and 2018 Guinea Demographic and Health Survey (GHDS), collected between June and October 2012 and February to June 2018, respectively. The GHDS uses a two-stage cluster design, with enumeration areas as the first stage sampling units. The enumeration units were selected proportionally according to their size. A fixed number of households was randomly chosen from a full list within each enumeration unit. All women aged 15–49 in the selected households were interviewed. All participants provided informed consent, and data were de-identified prior to the researchers’ access. Only women who had given birth in the previous year were included in the analysis. This was to ensure that the included participants responding in 2018 had given birth after the Ebola outbreak. This was also to minimize variability among respondents in 2012 and maintain consistency with the 2018 data used. In 2012, 9142 women between the ages 15 and 49 were interviewed. Of those women, 2667 had given birth within the last year and were included in the current study. In 2018, 8000 women between the ages 15 and 49 were interviewed. Of those women, 3219 had previously given birth within the last five years and were eligible for analysis in the current study. Only observations from women who had complete data for antenatal care outcomes were included in the final analysis. The total sample size from 2012 was 1369 and was 1590 from 2018. There were significant differences between the included and excluded participants in 2012. A greater proportion of included women attended at least four ANC visits, saw a healthcare provider for ANC, received ANC in a health facility, were literate, achieved secondary education or higher, and worked for pay. A greater proportion of women not included in 2012 were in polygamous marriages; the mean age was higher among those excluded (M = 28.5, SD = 6.6). There were also significant differences between the included and excluded participants in 2018. A greater proportion of excluded participants in 2018 initiated ANC in the first trimester, used modern contraception and worked for pay. The mean age was higher in the excluded group (M = 29.6, SD = 7.6). Despite the differences between the included and excluded samples, a total number of ANC components could not be generated for the excluded sample, justifying their removal from the analysis.

#### Outcome Variable

All participants were asked questions regarding ANC for their most recent birth in the last five years. Women provided information about their antenatal care and what components of care they received. Possible components of ANC visits included blood pressure measurement, urine testing for the detection of bacteriuria and proteinuria, blood tests for syphilis and anemia, and the provision of iron supplementation, intestinal parasite drugs and tetanus toxoid injections. There was not a question regarding health education during pregnancy; therefore, we examined the quality of ANC using six indicators instead of the traditional seven. The number of ANC components received was the dependent variable (0–6). 

### 2.2. Covariates

This study was guided by the framework used in Joshi et al. [22] and Blackstone [31], examining associations between aspects of women’s empowerment and quality ANC using Demographic and Health Surveys (DHS) data. Based on the variables included in their analysis, as well as the previous research on correlates of ANC initiation and number of visits, the following variables were considered for their potential association with the outcome variable: place of residence, whether the pregnancy was intended, birth control use, location of ANC, ANC provider, number of ANC visits, timing of ANC initiation, education, parity, maternal age, wealth, media exposure, employment, payment for employment, decision-making power over healthcare, attitudes toward intimate partner violence (IPV), polygamy in the marriage, and literacy. 

Place of residence was coded as urban or rural based on population density criteria at the time the survey was conducted [56]. Pregnancy intention was dichotomized as “yes” or “no.” Contraceptive use was categorized in one of three ways: no birth control, traditional methods or modern methods. Location of ANC was collapsed into three categories: home, NGO facility or health facility. Initially, ANC provider had six categories, which were dichotomized as receiving care from a skilled healthcare provider versus receiving care from a non-healthcare provider. Number of ANC visits was collapsed into two categories: attended 4+ visits or attended less than 4 visits. Timing of ANC initiation was dichotomized, indicating if care was initiated during the first trimester or later. Education was categorized as no education, primary education or secondary or higher based on the highest year of education received by the participant. Parity was assessed by women self-reporting the number of previous births they had had. Maternal age was self-reported in years. The wealth quintiles established by the DHS were used as a proxy of socioeconomic status and were dummy coded. Media exposure assessed the frequency of exposure to magazines/newspaper, television and radio, respectively. These were dichotomized as exposed to form of media at least once per week and exposed to form of media less than once per week. Employment status was determined by whether the participant worked in the last twelve months. Employment for pay was categorized as no work, work for no pay, in-kind payment only, and cash payment for work. Decision making was assessed using a composite measure including decisions regarding healthcare, household purchases, visiting family and friends and who spends the respondent’s earnings. Each decision was also examined independently for its relationship with ANC. Responses were dichotomized and included husband only or woman is involved in decision. Attitudes toward IPV were assessed by asking women whether a husband is justified beating his wife in each of the following scenarios: if she burns food, if she withholds information from him, if she neglects the children, if she argues with him, and if she refuses to have sex with him. Higher scores are indicative of a greater acceptance of IPV (α = 0.86). Participants were asked how many wives their male partners had. This was coded into two categories: monogamous marriage or polygamous marriage. Finally, literacy was a binary variable with two categories: unable to read or able to read parts of/whole sentences. Literacy strictly measured functional reading ability and was not a proxy for health literacy. 

### 2.3. Statistical Analysis

The analyses used sampling weights and were adjusted by clustering and stratification of the sample design. Descriptive statistics, including mean and standard deviations and proportions, were obtained. Potential variables of interest were examined in bivariate analyses with number of ANC components. A cut-off of *p* < 0.25 was used for bivariate analyses, as more traditional values (e.g., *p* < 0.05) can fail to detect important covariates [57]. Adjusted odds ratios (aOR) and 95% confidence intervals (CI) were calculated using multiple logistic regression models examining associations between covariates and number of ANC components received. The Hosmer–Lemeshow statistic was used to assess model fit. The *p* values for all models were non-significant, indicating no evidence of poor fit. Statistical significance in the final regression model was determined at *p* < 0.05. All analyses were conducted using STATA 15 (StataCorp. College Station, TX, USA).

## 3. Results

A total of 2958 women were included in the final analysis (mean age = 27.2; SD = 7.0); 1368 from 2012 (mean age = 27.1; SD = 7.1) and 1590 from 2018 (mean age = 27.3; SD = 6.9). Descriptive statistics and sample demographics are presented in Table 1.

In 2012, 19.6% (*n* = 224) received all measured components of ANC; in 2018, 32.0% (*n* = 509) received all measured components, which was a significantly greater proportion than in 2012, χ^2^ (1) = 96.4; *p* < 0.001. Women in 2012 received, on average, 3.9 (SD = 1.6) of the recommended components of ANC visits, compared to 4.8 (SD = 1.3) in 2018, which was significantly greater than the average in 2012, *t*(2956) = 18.3; *p* < 0.001. 

The final variables included in the analysis were attendance of four or more ANC visits, timing of first ANC visit, ANC provider, ANC location, contraception use, exposure to magazines, radio and television, literacy, work for pay, residence, involvement in healthcare decision-making, endorsement of IPV attitudes, education, wealth, and polygamous marriage. The logistic regression model was statistically significant, χ^2^(24) = 916, *p* < 0.001. The model explained 29.3% (Nagelkerke R^2^) of the variance in the number of ANC components received. Controlling for all covariates, the adjusted results showed that women in 2018 had higher odds of receiving more components of ANC compared to women in 2012 (aOR = 3.90; *p* < 0.001, CI, 3.27, 4.64). Receiving at least four ANC visits (aOR = 1.76, *p* < 0.001, CI, 1.50, 2.06) and attending ANC with a health provider (aOR = 1.56, *p* < 0.001, CI, 1.31, 1.85) were associated with greater odds of receiving more of the necessary ANC components. In comparison with the lowest wealth quintile, women in the middle (aOR =1.66, *p* < 0.001, CI, 1.28, 2.16), rich (aOR = 1.95, *p* < 0.001, CI, 1.35, 2.83) and richest (aOR = 1.20, *p* < 0.001, CI, 1.03, 1.40) groups had greater odds of receiving more necessary ANC components. Living in rural regions was associated with lower odds of receiving higher numbers of ANC components (aOR = 0.47, *p* < 0.001, CI, 0.37, 0.60). See Table 2. 

Several aspects of women’s empowerment were significantly linked with receiving a great number of ANC components, such as involvement in decisions regarding personal healthcare (aOR = 1.20, *p* < 0.01, CI, 1.03, 1.40). Those involved in polygamous marriages had decreased odds of receiving ANC components (aOR= 0.86, *p* < 0.01, CI, 0.74, 0.99). Women who used traditional contraception (aOR = 0.35, *p* < 0.001, CI, 0.25, 0.49) or no contraceptives (aOR = 0.58, *p* < 0.001, CI, 0.86, 0.93) had lower odds of receiving greater numbers of ANC visit components compared to women using modern contraceptives. Women who did not work received more ANC components compared to women who worked for case payment (aOR = 1.42, CI, 1.00, 2.02). Finally, women endorsing a greater number of IPV scenarios were less likely to receive components of ANC (aOR = 0.89, CI, 0.86, 0.93).

## 4. Discussion

The results of this study revealed that although the proportion of women receiving at least four ANC visits decreased significantly between 2012 (59.5%) and 2018 (41.4%), the proportion of women receiving all recommended components increased from 19.6% to 32% within the same time fame. In addition, the mean number of components received increased from 3.9 (2012) to 4.8 (2018). This further points to the need to focus on the quality of ANC care rather than the number of visits received by pregnant women. Fewer visits that include more recommended components of care may be better than more visits that cover less components. Guinea has attempted to improve the provision of ANC to address the high maternal mortality rate. The Ebola outbreak of 2014 could have been a barrier to achieving these goals due to its impact on the healthcare system. Guinea’s improved provision of quality ANC care during this time may reflect the commitment to addressing women’s empowerment.

### 4.1. Women’s Empowerment and Receipt of ANC Care

#### 4.1.1. Decision-Making

Involvement in decisions regarding personal healthcare was associated with receiving a greater number of ANC components. Though the majority (66.4%) of women indicated their husbands were the sole decision-maker for healthcare, those who were also involved in decision-making were more likely to receive a greater number of quality components than those that did not. A number of previous studies have found women’s decision-making power to be positively associated with the number of ANC visits they received [22,58,59,60,61], though Ghose et al. [62] found that this difference was mediated by rural versus urban residence. Almost all these studies operationalized decision-making as a composite of a woman’s ability to make decisions about household purchases, visiting family, and personal healthcare. In addition, they measured the utilization of ANC care as the number of visits and did not include a measure of the quality of care as indicated by the number of components covered. While the traditional DHS composite measure of decision making was not statistically significant in the bivariate analyses in the present study, women’s healthcare decision-making was. Given its importance in women’s health and empowerment, and its previous demonstrated links to autonomy [63,64,65], it was included in the present study and was a significant predictor of quality ANC. This further supports the literature demonstrating the importance of women’s empowerment and autonomy over her healthcare in promoting positive health outcomes. 

#### 4.1.2. Polygamous Marriages

In our study, women whose husbands had other wives were less likely to receive quality ANC care than those who were not in polygamous marriages, in-line with other research that found a relationship between polygamy and non-use of ANC [66,67,68]. Women in polygamous relationships may have less decision-making power and less control over money than those in monogamous relationships [69]. However, this association may not be consistent across populations, as studies in Burkina Faso and Nigeria found that polygamous marriage was not associated with decreased ANC use [70,71], but rather other factors were, such as distance from a health facility [70]. 

#### 4.1.3. Use of Contraception

Our study found that women who used no contraception or traditional methods had lower odds of receiving ANC components compared to women using modern contraception. Do and Hotchkiss [72] analyzed data from Kenya and Zambia, and found that women who had higher ANC service intensity scores (a composite variable measuring the number, timing and components of ANC visits) consistently adopted modern contraceptives earlier and at a higher rate after giving birth. Post-partum family planning counseling is often provided within the context of ANC visits. If providers are discussing family planning as a component of ANC, they may be more likely to also provide other components of quality ANC. Do and Hotchkiss [72] also found that those who had ever used modern contraception before conception of their last child used ANC services more intensively than others did. Afulani [73] hypothesized that women who had ever used modern contraception potentially had greater familiarity with the health system and sought maternity providers that would provide higher quality care. Women who use modern contraception and seek quality care during the preconception period may experience higher self-efficacy, which carries over into the antenatal period, impacting the quality of their ANC care. However, Joshi and colleagues [22] found that in Nepal, the use of modern family planning was not associated with an increased number of ANC visits, and was associated with a decreased quality of ANC. There may be additional contextual factors that play a role in this relationship that need to be explored in future research.

#### 4.1.4. Literacy and Access to Media

In this sample, literacy was not a significant predictor of quality ANC, however there are several studies noting its importance in pregnancy care and outcomes. One study [73] looked at both the usage and components of ANC visits, and found that those who were literate were also more likely to have quality ANC care. In addition, studies in South Sudan and Ethiopia found that the non-use of any ANC services was significantly higher among illiterate women than those who could read [66,67]. One qualitative study in Northern Tanzania found that illiteracy limits healthcare-seeking because women could not read their healthcare cards or other public health messaging, and often did not speak the language used by skilled healthcare providers [74]. Interestingly, a recent Ethiopian study found that the association between literacy and the number of ANC visits decreased between DHS data collections in 2005 and 2011, and disappeared altogether in 2016, with similar trends found in partner’s education level [75]. This may be reflective of health systems that are successful in removing barriers to care and increasing women’s autonomy despite low educational attainment. Because the majority of the sample (82%) did not have basic literacy skills, there may not have been sufficient variation in the sample to see the effect of literacy on quality ANC. Despite the lack of significant literacy in this study, it may be a notable effect in the future as women’s empowerment initiatives in Guinea continue.

Beyond basic literacy, several studies in Ethiopia and Nigeria have found that the frequent exposure to media (television, radio, magazines) had a positive influence on the likelihood of ANC use [60,76,77,78,79]. Mass media plays a key role in informing expectant mothers about the importance of ANC to reduce pregnancy risks and improve health outcomes, particularly among women with low educational attainment [80,81]. While these studies created a composite variable for mass media use, our study looked at television, radio and magazine use separately, and did not find any significant differences in the number of ANC components that women received or the use of these media sources. The impact of radio and television use may be becoming less significant given that over the last two decades they have become ubiquitous in many sub-Saharan countries [82]. Further, with the growth of smart phone use and access to the internet, magazine use may be declining and may not be a strong indicator of empowerment. 

#### 4.1.5. Socio-Economic Status and Employment

Our study found that as a women’s socio-economic status increased, she was likely to receive more components of quality ANC. Similarly, a study from Bangladesh found that mothers in middle and richer groups were 1.3 and 1.5 times more likely to receive recommended ANC components relative to women in the poorest groups [83]. Household wealth also positively influences attendance of at least one ANC visit, with women of the highest wealth quintile attending on average four ANC visits more than those in the lowest [84]. Socio-economic status is a significant predictor of health-seeking behavior and the utilization of ANC among women. Financial resources are an enabling factor that allow women to pay for maternity services where they are not provided for free, as well as other related non-medical costs, such as travel [85,86]. However, in an Ethiopian study, Ousman and colleagues [75] found that this disparity was decreasing over time as systemic steps were being undertaken to remove barriers to accessing and improving maternal healthcare.

In our study, women who worked for pay or in-kind donations were more likely to receive quality ANC compared to those who did not. Afulani and colleagues [87] also found that employment was associated with higher-quality ANC. Atinga [88] found that women employed in the private sector were twice as likely to perceive that they received quality ANC compared to those who were not. The previous literature also shows a strong relationship between number of ANC visits and employment [58,60,75,89,90]. However, one study from Nigeria found that there was no difference between users and non-users of ANC by employment status [91], though the researchers did not look at the type of employment (formal/informal, paid/in-kind). Employment in general, as well as the type of employment, is associated with education level, access to money, and increased mobility, which increases the likelihood of accessing ANC [90]. 

### 4.2. Limitations and Strengths

There are limitations to this study that warrant discussion. First, the data were self-reported in a retrospective study and subject to recall and response bias. Additionally, women’s responses to the empowerment questions were reflective of when they completed the survey, not when they were pregnant. To mitigate these concerns, only women who had given birth in the year prior to data collection were included. Second, we did have to exclude a large proportion of the sample due to incomplete data, and there were significant differences in some indicators between the women included in the analysis and those not. Women included from 2012 showed more characteristics linked with greater empowerment (e.g., literacy, number of ANC visits, education, etc.) than women who were excluded. This suggests that in 2012, aspects of empowerment were linked with documentation of ANC. Thus, the statistics for number of ANC components received and whether quality care was given may be underestimations. In 2018, the samples differed on fewer variables; however, conversely, those excluded in 2018 were more likely to initiate ANC in the first trimester, use modern contraception, and work for pay. While these differences are notable, the outcome variable of interest was number of ANC components received and including participants with missing data for any of the indicators would have led to inaccurate findings. 

Another limitation is that some women included in the analysis may not have known, or could not remember, what components of ANC they received. As a result, women may have reported receiving certain components due to social desirability bias when they did not or may have reported not receiving certain components when they did. It is possible that the proportion of women receiving quality ANC is over- or underestimated. Moreover, the variable measuring place of ANC did not represent all the different types of health facilities women may have attended; the categories were groups intended to facilitate the interpretability of results. Likewise, the ANC provider may change throughout pregnancy, and the provider reported at the time of data collection may not have been the provider throughout the entire pregnancy. Finally, women’s empowerment is a complex concept that could be measured by variables other than the ones included in this study. To avoid oversaturating the regression model and make results more meaningful for practical application, only variables significant in bivariate associations were included, removing certain variables from the final analysis that are significant in other studies. For instance, women’s decision-making power, often conceptualized as a composite score of decision power in healthcare, household purchases and social interactions, was not significant in bivariate analyses for the current study and was omitted from the final analysis. Therefore, there are likely other factors that influence the receipt of quality ANC not explored by the present study, that should be considered in the future.

Despite these limitations, this study has notable strengths. We used data from a population-based survey with a large, representative sample. The Demographic and Health Surveys were conducted using rigorous standardized protocols to ensure adequate representation of the country and the quality of the data. Few studies explore the relationship between empowerment and participation in ANC; most that do examine the timing and number of ANC visits, rather than components of care that were included in the visits. Further, this is the only study to our knowledge that compares national data in Guinea on indicators of quality ANC and empowerment. Examining quality ANC is Guinea is important, especially recognizing the critical period wherein national efforts to improve women’s empowerment also align with a major disease outbreak such as Ebola. This study further demonstrates the importance of efforts to improve women’s empowerment in Guinea and improve pregnancy outcomes.

## 5. Conclusions

While the proportion of women receiving quality ANC increased from 19.6% in 2012 to 32% in 2018, there remains a need to focus on promoting the quality of ANC, not just the quantity of visits and timing of visits. However, women who initiated care in the first trimester and attended four or more visits were more likely to receive quality care, suggesting these aspects of ANC should remain a priority. Though women in 2018 attended on average fewer ANC visits than women in 2012, they had a greater chance of receiving quality care. In examining maternal and child health outcomes over time, this offers interesting information, suggesting that efforts to improve antenatal care in Guinea have been successful, even if it this not reflected in a greater number of clinic visits. 

Further, there was concern over the growing need for quality ANC as a result of the Ebola outbreak in 2014 [92], and the potential for the outbreak to jeopardize maternal and child healthcare development. The data from this study suggest efforts to improve healthcare for pregnant women are coming to fruition, and the benefits of these initiatives are reflected in the number of women accessing more of the necessary components of ANC. Disease outbreaks have far-reaching negative effects on access to quality care. However, more empowered women may have the self-efficacy to navigate complicated healthcare contexts to receive care. Despite the Ebola outbreak, the MMR in Guinea continued to improve from 747 deaths per 100,000 live births in 2010 to 699 in 2015 (just after the outbreak), with similar declines in infant mortality [93,94]. Given the experience of Guinea during the 2014 Ebola outbreak, and the ongoing COVID-19 pandemic, national efforts to increase access to healthcare and improve women’s empowerment should not be halted. 

The study also highlights the importance of women’s autonomy and empowerment, particularly healthcare decision-making, contraceptive use and economic empowerment. Increased autonomy and empowerment among women may encourage the increased use of maternal care services and prove useful in addressing maternal and child outcomes, as well as Guinea’s larger goals of addressing gender disparities. The relationship between empowerment and quality ANC care may be cyclical. Our study found that participants that were more empowered were more likely to receive quality ANC care, whereas other studies have found that quality patient-centered maternity care (such as group ANC appointments) could further empower pregnant women, as it strengthens connections to healthcare providers and other women [95].

## Figures and Tables

**Table 1:** Descriptive Statistics.

Variable		2012	2018	Test for Differences	Total
		*N*(%) or M(SD)	*N*(%) or M(SD)	Χ^2^ or *t*	*N*(%) or M(SD)
Number of ANC components received		3.98 (1.6)	4.8 (1.3)	*t* = 18.3 ***	4.3 (1.5)
Received all ANC components		224 (19.6)	509 (32.0)	Χ^2^ = 96.4 ***	733(24.9)
Attended 4+ ANC visits		865 (63.4)	616 (40.0)	Χ^2^ = 159.7 ***	1481(51)
ANC visit in 1st trimester		616 (45.1)	488 (31.0)	Χ^2^ = 62.7 ***	1104 (37.5)
ANC with healthcare provider		927 (67.8%)	1219 (76.7%)	Χ^2^ = 28.9 ***	2146 (72.6%)
ANC location				Χ^2^ = 329.3 ***	
	Home	13 (0.01)	0 (0)		13 (0.4)
	Non-health facility	65 (4.8)	537 (33.8)		602 (20.4)
	Healthcare facility	1289 (94.3)	1053 (66.2)		2342 (79.2)
Contraception				Χ^2^ = 157.9 ***	
	None	1293 (94.5)	1412 (88.8)		2705 (91.5)
	Traditional	64 (4.6)	13 (0.01)		77 (2.6)
	Modern	11 (0.01)	165(10.4)		176 (5.9)
Magazine 1+ per week		30 (2.2)	45 (2.8)	Χ^2^ = 1.2	75 (2.5)
Radio 1+ per week		501 (36.6)	518 (32.6)	Χ^2^ = 5.0 *	1019 (34.5)
Television 1+ per week		234 (17.1)	297 (18.6)	Χ^2^ = 3.7	665(22.5)
Can read parts of/complete sentences		234 (17.1%)	297 (18.7%)	Χ^2^ = 1.3	531 (18.0%)
Paid employment				Χ^2^ = 82.3 ***	
	No work	310 (22.7)	469 (29.5)		779 (26.3)
	No pay	309 (22.6)	495 (31.1)		804 (27.2)
	In-kind pay only	89 (0.07)	40 (2.5)		129 (4.4)
	Cash payment	660 (48.5)	586 (42.1)		1246 (42.1)
Husband has other wives		517 (41.2)	578 (38.4)	Χ^2^ = 2.3	1095 (39.6)
Decision maker for healthcare				Χ^2^ = 16.8 ***	
	Husband only	878 (70.5)	943 (63.0)		1821 (66.4)
	Woman has a say in decision	943 (29.5)	553 (37.0)		921 (33.6)
Average number of decisions		0.9 (0.8)	1.87(1.7)	*t* = 18.9 ***	1.4 (1.5)
Average number of endorsed IPV scenarios		3.8 (1.5)	2.6 (1.9)	*t* = 17.2 ***	3.2 (1.8)
Age		27.1(7.1)	27.3(6.9)	*t* = 0.8	27.2 (7.0)
Urban residence		449 (32.8)	478 (30.1)	Χ^2^ = 2.6	927 (31.3)
Highest level of education				Χ^2^ =2.8	
	None	986 (72.1)	1159 (72.9)		2415 (72.5)
	Primary	204 (14.9)	212 (13.3)		416(14.1)
	Secondary	178 (13.0)	219 (13.8)		397 (13.4)
Wealth quintile				Χ^2^ = 6.3	
	Poorest	276 (20.2)	364 (22.9)		640 (21.6)
	Poor	266 (19.4)	328 (20.6)		594 (20.1)
	Middle	297 (20.4)	327 (20.6)		606 (20.5)
	Richer	323(23.6)	342 (21.5)		665 (22.5)
	Richest	224 (16.4)	229 (14.4)		453 (15.3)
Total		1368	1590		2958

* *p* < 0.05; *** *p* < 0.001.

**Table 2:** Results from adjusted logistic regression models.

Variable	aOR	Std. Error	95% CI	
*Time*				
2012				
2018	3.90 ***	0.35	3.27	4.64
*Timing of first ANC visit*				
After first trimester ^a^				
During first trimester	1.15	0.09	0.98	1.34
*Number of ANC visits*				
<4 ^a^				
≥4	1.76 ***	0.14	1.50	2.06
*Region*				
Urban ^a^				
Rural	0.47 ***	0.06	0.37	0.60
*Decision on healthcare*				
Husband only ^a^				
Woman involved	1.20 *	0.09	1.03	1.40
*Wealth*				
Poorest ^a^				
Poor	1.21	0.13	0.97	1.50
Middle	1.66 ***	0.22	1.28	2.16
Rich	1.95 ***	0.37	1.35	2.83
Richest	1.20 **	0.09	1.03	1.40
*Contraception*				
Modern methods ^a^				
Traditional methods	0.35 ***	0.06	0.25	0.49
No contraceptives	0.58 *	0.17	0.33	1.01
*IPV*	0.89 ***	0.02	0.86	0.93
*Magazine access*				
<1 time per week ^a^				
≥1 time per week	1.23	0.36	0.69	2.20
*Radio access*				
<1 time per week ^a^				
≥1 time per week	1.10	0.09	0.94	1.29
*TV access*				
<1 time per week ^a^				
≥1 time per week	1.06	0.12	0.84	1.32
*Works for pay*				
Cash payment ^a^				
In-kind payment	0.91	0.09	0.76	1.10
Works without pay	0.93	0.08	0.78	1.11
Does not work	1.42 *	0.26	1.00	2.02
*Education*				
No education ^a^				
Primary education	0.76	0.52	0.20	2.91
Secondary education	1.06	0.42	0.49	2.29
*Literacy*				
Cannot read ^a^				
Can read some	1.32	0.25	0.91	1.91
*Husband has other wives*				
No ^a^				
Yes	0.86 *	0.06	0.74	0.99
*Location of ANC*				
Non-health facility/home ^a^				
Healthcare facility	1.08	0.10	0.90	1.30
*ANC provider*				
Non-healthcare provider ^a^				
Healthcare provider	1.56 ***	0.14	1.31	1.85

^a^ Reference category * *p* < 0.05; ** *p* < 0.01; *** *p* < 0.001.
